# A Novel Prognostic Score Including the CD4/CD8 for AIDS-Related Lymphoma

**DOI:** 10.3389/fcimb.2022.919446

**Published:** 2022-07-06

**Authors:** Juanjuan Chen, Xuewu Liu, Shanfang Qin, Guangjing Ruan, Aili Lu, Jinxin Zhang, Yihua Wu, Zhiman Xie, Jie Peng

**Affiliations:** ^1^ Department of Infectious Diseases, Nanfang Hospital, Southern Medical University, Guangzhou, China; ^2^ Department of Neurology, Nanfang Hospital, Southern Medical University, Guangzhou, China; ^3^ Guangxi AIDS Diagnosis and Treatment Quality Control Center, Longtan Hospital of Guangxi Zhuang Autonomous Region, Liuzhou, China; ^4^ Guangxi AIDS Clinical Treatment Center, The Fourth People’s Hospital of Nanning, Nanning, China

**Keywords:** AIDS-related lymphomas, CD4/CD8 ratio, novel prognostic score, independent prognostic parameter, inflammatory marker

## Abstract

**Background:**

A simple and clinically applicable prognostic scoring system for AIDS-related lymphoma (ARL) in the era of combination antiretroviral therapy (cART) is needed to better stratify patients’ risks and to assist in the decision-making of therapeutic strategies.

**Methods:**

We conducted a retrospective multicenter cohort study in 138 primary ARL patients over an 8-year period from 2013 to 2020. Survival curves were estimated using the Kaplan–Meier method. Univariate and multivariate Cox proportional hazard models were performed to identify the association between patient-, lymphoma-, and HIV-specific variables with progression-free survival (PFS) and overall survival (OS). The incremental prognostic value of novel inflammatory biomarkers in the International Prognostic Index (IPI) was evaluated by comparing the receiver operating characteristic (ROC) curves, the concordance index (C-index), and the integrated Brier score (IBS).

**Results:**

The median age was 49.14 ± 14.20 (range 18–79) years, 81.9% were men, and the median follow-up was 44.94 (95% CI = 37.05–52.84) months. The 3-year OS and PFS were 39.4% (95% CI = 16.3–21.2) and 38.7% (95% CI = 14.5–19.7), respectively. We found that age, extranodal sites, bulky mass, CD4 T-cell counts, CD4/CD8 ratio, and hypoalbuminemia were associated with OS (all *P* < 0.05) at both univariate and multivariate analyses. Of the new inflammatory markers, only the CD4/CD8 ratio was an independent prognostic parameter of OS and PFS. A lower CD4/CD8 ratio was strongly associated with adverse clinical factors, including older age, advanced Ann Arbor stage, more extranodal sites, elevated erythrocyte sedimentation rate, prior history of HIV, higher red cell distribution width ratio, hypoproteinemia, and emaciation. When the CD4/CD8 ratio was added to the IPI, the composite HIV-IPI score showed significantly better discrimination than IPI alone [AUC (95% CI): HIV-IPI, 0.83 (0.77–0.89) vs. IPI, 0.72 (0.70–0.85)]. The HIV-IPI model provided good predictive performance [C-index (95% CI): HIV-IPI, 0.82 (0.81–0.83) vs. IPI, 0.75 (0.73–0.77), *P* < 0.001] and a satisfactory calibration function.

**Conclusions:**

The CD4/CD8 ratio, an inexpensive and readily available marker, is a powerful independent prognostic parameter in patients with ARL. Furthermore, when the CD4/CD8 ratio is used in combination with IPI, it increases prognostic ability. The useful prediction of expected outcomes in ARL can inform treatment decisions.

## Introduction

AIDS-related lymphoma (ARL) remains a leading cause of morbidity and mortality for people living with HIV (PLWH), even in the era of combination antiretroviral therapy (cART) (NOY, 2019; Re et al., 2020). Approximately 70%–90% of patients with ARL are diagnosed with diffuse large B-cell lymphoma (DLBCL) and Burkitt lymphoma (BL) with phenotypically and genetically heterogeneous and aggressive diseases, compared to HIV-uninfected patients (Yarchoan and Uldrick, 2018; Kimani et al., 2020). Safer and better tolerated HIV-directed therapies can reduce the impact of HIV-related factors on outcomes and allow intensive immunochemotherapy for ARL individuals in the cART era (Barta et al., 2015; NOY, 2019). Therefore, the outcome of patients with ARL who receive rituximab-containing regimens has improved in recent years in economically developed countries (Schommers et al., 2018). However, patients with ARL from countries with limited resources often lack adequate treatment. Furthermore, prognostic assessment for patients with ARL remains poor, representing an unmet clinical need.

Three scoring systems incorporating simple clinical parameters constitute crucial parts of immunocompetent patients with aggressive evaluation and management of non-Hodgkin lymphoma B cells: the International Prognostic Index (IPI), age-adjusted IPI (aaIPI), and the National Comprehensive Cancer Network IPI (NCCN-IPI) (Ruppert et al., 2020). However, none of these clinical risk scores can precisely identify the ARL patient subgroup. Barta et al. (2014) defined a prognostic score for ARL by assigning weights to the HIV score (composed of CD4 count, viral load, and prior history of AIDS). However, it is still controversial whether HIV characteristics, such as viral load and CD4 count, influence prognosis in contemporaneously treated ARL (Barta et al., 2015; Ramos et al., 2020; Alderuccio et al., 2021). Therefore, a clinically applicable risk stratification system is required for HIV populations with ARL. In addition, the identification of new independent prognostic factors can help to stratify risk more accurately.

In recent years, attention has focused on novel inflammatory markers such as the CD4/CD8 ratio, hemoglobin to red cell distribution width ratio (Hb/RDW), platelet to lymphocyte ratio (PLR), and lymphocyte to monocyte ratio (LMR), which emerged as part of the initial evaluation strategy to identify tumor progression and therapeutic response (Serrano-villar et al., 2020; Caby et al., 2021; Koh et al., 2022). These hematological indices can be easily abstracted from the complete blood cell count and are prognostic values in HIV and malignant neoplasms (Keane et al., 2015; Mcbride and Striker, 2017). However, the role of such factors in the prognosis of ARL remains unclear. Therefore, the integration of HIV and inflammatory characteristics into the IPI could better characterize risk stratification. The objectives of this study were to evaluate independent clinical risk factors and build a novel, easily applicable, and better risk-stratified prognostic model for patients with ARL.

## Materials and Methods

### Patients

This was a retrospective multicenter cohort study conducted over an 8-year period from 2013 to 2020 in three hospitals in China, namely, the Nanfang Hospital Affiliated with Southern Medical University, the Fourth People’s Hospital of Nanning, and the Longtan Hospital of the Guangxi Zhuang Autonomous Region. We performed a pooled analysis of existing databases of 138 patients with primary AIDS-related lymphoma with available information. The pathological diagnosis of lymphoma was based on the 2008 World Health Organization (WHO) classification.

### Data Collection and Endpoints

Information on patient demographics, diagnosis, treatment choice, standard clinical and laboratory parameters, and details on outcome was collected for the analysis. The expression of CD10, MUM1, and BCL6 was used to determine the germinal center B-cell (GCB) phenotype using the Hans algorithm (Hans et al., 2004). The parameters relevant to the new inflammatory markers were evaluated, including the CD4/CD8 ratio, Hb/RDW, PLR, and LMR. Data were presented as percentages for categorical data and medians for continuous data. The main endpoints of this study were progression-free survival (PFS) and overall survival (OS). PFS was defined as the time from diagnosis to progression, relapse, or death from any cause. OS was defined as the time from diagnosis until death from any cause.

### Construction and Validation of the HIV-IPI

Cox proportional hazard regression models were applied for univariate and multivariate analyses to confirm significant predictors. Novel inflammatory biomarkers with *P <*0.05 were considered statistically significant, which were incorporated into the IPI. The evaluation of the incremental prognostic value of the predictors was performed by comparing the receiver operating characteristic (ROC) curve, the concordance index (C index), and the integrated Brier score (IBS) between the IPI and the HIV-IPI risk score. A calibration curve was derived based on regression analyses to show the concordance between the predicted and observed probabilities for survival.

### Statistical Analysis

Survival curves were calculated using the Kaplan–Meier method and compared using the log-rank test, with 95% confidence interval (CI). The optimal cutoff values of the biomarkers were calculated using the ROC curve analysis and the Youden index. Univariate and multivariate analyses were performed using the Cox proportional hazard model. The hazard ratio (HR) and 95% CI were used to summarize the association between variables and survival. Linear correlation analysis and Student’s *t*-test were used to analyze the relationship of the CD4/CD8 ratio with other factors. The distributions of clinical characteristics between the different groups were performed using the Pearson chi-square, Fisher’s exact test, or continuity correction. All statistical analyses were performed with SPSS 25.0 (IBM Corp., Armonk, NY, USA). All statistical tests were two-sided and significance was defined as *P*-value <0.05.

## Results

### Clinical Characteristics and Survival

**Figure 1:**
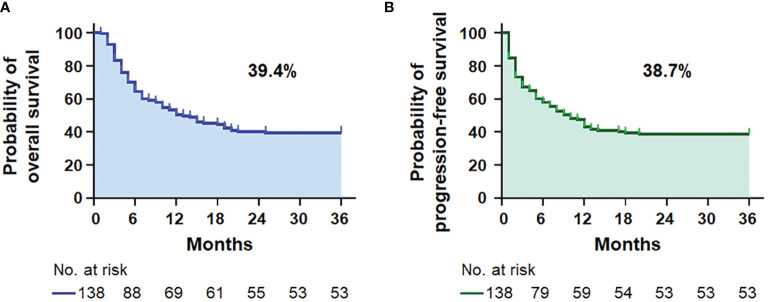
Survival analysis. Kaplan–Meier survival curves for the 3-year overall survival **(A)** and progression-free survival **(B)** in 138 patients with AIDS-related lymphoma (*n* = 138).

The demographic and clinical characteristics of 138 patients with AIDS-related lymphoma with complete data in our cohort are summarized in **Table 1**. The median age was 49.14 ± 14.20 (range 18–79) years, 81.9% were men, and the median follow-up was 44.94 (95% CI = 37.05–52.84) months. DLBCL was the most common subtype identified (86.2%, **Supplementary Figure 1A**), and AIDS-associated DLBCL had enrichment for the GCB subtype (62.2%, **Supplementary Table 1**). According to the IPI score, 37 (26.8%) patients with ARL were aged >60 years old, 106 (76.8%) presented elevated LDH, 54 (39.1%) had Eastern Cooperative Oncology Group performance status (ECOG PS) >1 site, 61 (44.2%) had advanced disease (Ann Arbor stage III/IV), and 35 (25.4%) had extranodal disease >1 (**Table 1**). Moreover, there were 50 patients (36.2%) in the low-risk group, 37 (26.8%) in the low-intermediate-risk group, 26 (18.8%) in the high-intermediate risk group, and 25 (18.2%) in the high-risk group. B symptoms were observed in 22 (15.9%) patients and bulky mass (>7 cm) was found in 61 (44.2%) patients. A history of HIV was documented in 23.9% of the patients. The mean CD4 T-cell count and CD4/CD8 ratio were, respectively, 221.19 ± 172.18 cells/µl (range 3–1,089) and 0.37 ± 0.27 (range 0.03–1.69). Of the 138 patients, 87.0% received cART. Most of them chose a free cART regimen with tenofovir, lamivudine, and efavirenz (60.1%, 83/138), and only 12 patients (8.7%) included integrase inhibitor-based therapy (**Supplementary Table 1**). The 3-year OS and PFS rates were 39.4% and 38.7% for patients with ARL, respectively (**Figure 1**). The median OS was 13.0 (95% CI = 7.3–18.7) months for the ARL cohort (**Supplementary Figure 1B**). Treatments and results of ARL are summarized in **Supplementary Table 1**.

**Table 1:** The demographics and clinical characteristics of AIDS-related lymphoma (ARL) patients (*n* = 138).

Characteristics	DLBCL	BL	Other ARL	Total
**Sample size, *n* (%)**	119 (86.2)	9 (6.5)	10 (7.3)	138 (100)
**Age (range)**	48.69 ± 14.20 (18–77)	54.22 (41–68)	49.90 (27–79)	49.14 ± 14.20 (18–79)
**Male, *n* (%)**	97 (81.5)	6 (66.7)	10 (100)	113 (81.9)
**IPI factor, *n* (%)**				
** Age > 60 years**	30 (25.2)	3 (33.3)	4 (40.0)	37 (26.8)
** Elevated LDH**	93 (78.2)	8 (88.9)	5 (50.0)	106 (76.8)
** ECOG PS > 1**	50 (42.0)	3 (33.3)	1 (10.0)	54 (39.1)
** Stage III/IV**	54 (45.4)	4 (44.4)	3 (30.0)	61 (44.2)
** Extranodal sites > 1**	31 (26.1)	2 (22.2)	2 (20.0)	35 (25.4)
**IPI, *n* (%)**				
** Low**	40 (33.6)	4 (44.4)	6 (60.0)	50 (36.2)
** Low-intermediate**	34 (28.6)	2 (22.2)	1 (10.0)	37 (26.8)
** High-intermediate**	23 (19.3)	1 (11.1)	2 (20.0)	26 (18.8)
** High**	22 (18.5)	2 (22.2)	1 (10.0)	25 (18.2)
**E-involvement, *n* (%)**	70 (58.8)	5 (55.6)	6 (60.0)	81 (58.7)
**Bulky mass, *n* (%)**	21 (17.6)	5 (55.6)	5 (50.0)	61 (44.2)
**B symptoms, *n* (%)**	51 (42.9)	1 (11.1)	0 (0)	22 (15.9)
**Prior history of HIV, *n* (%)**	27 (22.7)	2 (22.2)	4 (40.0)	33 (23.9)
**LDH (range)**	802.60 ± 1,100.27 (153–9,213)	524.17 (216–811)	436.38 (116–1,974)	757.90 ± 1,039.08 (116–9,213)
**CD4 T cell (range)**	208.13 ± 168.58 (3–1,089)	280.78 (73–502)	322.90 (32–644)	221.19 ± 172.18 (3–1,089)
**CD4/CD8 (range)**	0.34 ± 0.24 (0.03–1.47)	0.56 (0.12–1.15)	0.50 (0.07–1.69)	0.37 ± 0.27 (0.03–1.69)
**PLT (range)**	248.18 ± 120.12 (22–635)	310.79 (128–587)	270.30 (22–575)	253.86 ± 123.55 (22–635)
**LMR (range)**	3.17 ± 2.58 (0.25–16.50)	2.36 (1.30–4.25)	4.21 (1.20–16.50)	3.19 ± 2.73 (0.25–16.50)
**PLR (range)**	251.81 ± 316.34 (5.60–3,140.00)	250.89 ± 131.60 (80–477)	209.82 ± 110.00 (5.20–383.00)	248.71 ± 296.85 (5.20–3,140.00)
**RDW ratio (range)**	0.99 ± 0.18 (0.66–1.73)	0.99 ± 0.22 (0.82–1.42)	0.98 ± 0.12 (0.82–1.26)	0.99 ± 0.18 (0.66–1.73)
**Hb/RDW (range)**	7.80 ± 2.50 (2.03–12.80)	7.84 ± 2.45 (4.28–11.05)	7.46 ± 2.17 (3.75–9.76)	7.95 ± 2.46 (2.03–12.80)
**ESR (range)**	52.30 ± 37.03 (2–160)	74.00 ± 39.96 (25–129)	54.89 ± 39.20 (6–138)	54.20 ± 37.53 (2–115)
**ALB (range)**	34.69 ± 7.71 (6–51)	35.19 ± 2.325 (33–40)	32.72 ± 6.16 (20–39)	34.58 ± 7.376 (6–51)
**BMI (range)**	21.59 ± 3.48 (12.11–35.88)	22.87 (18.37–25.97)	20.27 (17.53–25.10)	21.58 ± 3.39 (12.11–35.88)

DLBCL, diffuse large B-cell lymphoma; BL, Burkitt lymphoma; IPI, International Prognostic Index; LDH, lactate dehydrogenase; ECOG PS, Eastern Cooperative Oncology Group performance status; E-involvement, extranodal site involvement; PLT, platelet; LMR, lymphocyte to monocyte ratio; PLR, platelet to lymphocyte ratio; RDW ratio, red cell distribution width ratio; Hb/RDW, hemoglobin to red cell distribution width ratio; ESR, erythrocyte sedimentation rate; ALB, albumin; BMI, body max index.

### Independent Prognostic Factors for OS and PFS

**Figure 2:**
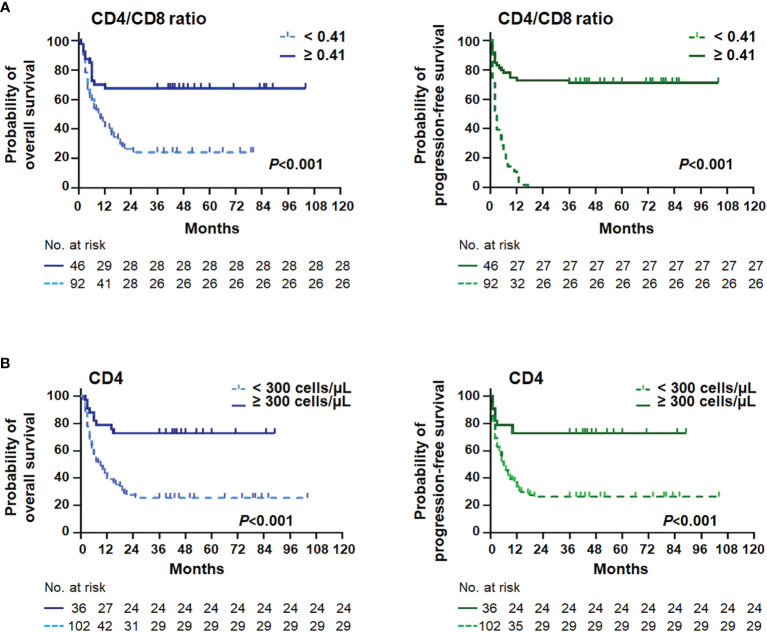
Survival curves stratified by the CD4/CD8 ratio and the CD4^+^ T cell. Kaplan–Meier curves and log-rank *P*-values of the CD4/CD8 ratio **(A)** and the CD4^+^ T-cell **(B)** stratifications for overall survival (left) and progression-free survival (right) in AIDS-related lymphoma patients (*n* = 138).

As shown in **Table 2**, the univariate analysis identified that the lymphoma and HIV-related parameters, including LDH, ECOG PS, Ann Arbor stage, bulky mass, B symptoms, IPI, aaIPI, NCCN-IPI, CD4 T-cell count, erythrocyte sedimentation rate (ESR), and albumin (ALB), had a significant impact on OS (all *P* < 0.05). In multivariate analysis, older age (HR = 1.721, 95% CI = 1.014–2.921, *P* = 0.044), more extranodal diseases (HR = 2.252, 95% CI = 1.061–4.781, *P* = 0.035), bulky mass (HR = 1.736, 95% CI = 1.037–2.908, *P* = 0.036), reduced CD4 T-cell count (HR = 2.378, 95% CI = 1.236–4.573, *P* = 0.009), and hypoalbuminemia (HR = 2.341, 95% CI = 1.436–3.820, *P* = 0.001) were identified as independent prognostic factors for inferior OS. Similarly, we found that greater extranodal diseases (HR = 2.232, 95% CI = 1.046–4.764, *P* = 0.038), bulky mass (HR = 1.713, 95% CI = 1.027–2.857, *P* = 0.039), reduced CD4 T-cell counts (HR = 2.328, 95% CI = 1.210–4.479, *P* = 0.011), and hypoalbuminemia (HR = 2.225, 95% CI = 1.362–3.636, *P* = 0.001) were independently associated with PFS in univariate and multivariate analyses (**Table 3**). The Kaplan–Meier analysis for OS and PFS showed that CD4 T-cell counts, ALB levels, and bulky mass were able to further stratify the risk of the high-risk and low-risk groups (**Figure 2** **and** **Supplementary Figure 2**), except prior history of HIV (**Supplementary Figure 5**).

**Table 2:** The factors associated with 3-year overall survival in AIDS-related lymphoma patients (*n* = 138).

Variables	Univariate analysis		Multivariate analysis	
	HR (95% CI)	*P*	HR (95% CI)	*P*
Age > 60	1.440 (0.906–2.289)	0.123	1.721 (1.014–2.921)	**0.044**
Male	1.135 (0.640–2.016)	0.664		
LDH > 250	2.315 (1.278–4.192)	**0.006**	1.877 (0.806–4.372)	0.145
ECOG PS > 1	1.895 (1.232–2.914)	**0.004**	1.429 (0.787–2.593)	0.241
Ann Arbor stage III/IV	1.548 (1.008–2.377)	**0.046**	1.660 (0.875–3.148)	0.121
Extranodal sites > 1	1.226 (0.763–1.970)	0.400	2.252 (1.061–4.781)	**0.035**
Bulky mass ≥ 7	1.833 (1.192–2.817)	**0.006**	1.736 (1.037–2.908)	**0.036**
B symptoms (present)	2.024 (1.199–3.416)	**0.008**	1.259 (0.621–2.554)	0.523
IPI		**<0.001**		
Low-intermediate	3.067 (1.685–5.583)			
High-intermediate	3.009 (1.584–5.715)			
High	3.289 (1.719–6.294)			
Prior history of HIV	1.084 (0.656–1.792)	0.753		
CD4 T cell < 300	3.021 (1.599–5.709)	**0.001**	2.378 (1.236–4.573)	**0.009**
CD4/CD8 ratio < 0.41	2.180 (1.292–3.678)	**0.004**	2.103 (1.156–3.827)	**0.015**
PLT ≥ 300	0.798 (0.512–1.244)	0.318		
LMR ≥ 3.65	0.622 (0.403–0.962)	**0.033**	0.666 (0.345–1.287)	0.227
PLR ≥ 250	1.810 (1.171–2.797)	**0.008**	1.088 (0.628–1.885)	0.763
RDW ratio ≥ 1.03	1.540 (0.993–2.391)	0.054		
Hb/RDW ≥ 8.5	0.532 (0.340–0.834)	**0.006**	0.746 (0.456–1.221)	0.244
ESR ≥ 80	1.951 (1.173–3.242)	**0.010**	1.154 (0.637–2.089)	0.637
ALB < 32	2.369 (1.529–3.669)	**<0.001**	2.341 (1.436–3.820)	**0.001**
BMI		0.172		
Overweight	1.260 (0.691–2.295)			
Emaciation	1.578 (0.819–3.039)			
aaIPI		**0.002**		
Low-intermediate	2.321 (0.891–6.048)			
High-intermediate	3.593 (1.385–9.324)			
High	5.053 (1.925–13.264)			
NCCN-IPI		**0.004**		
Low-intermediate	1.664 (0.698–3.965)			
High-intermediate	3.351 (1.403–8.004)			
High	3.252 (1.155–9.154)			

LDH, lactate dehydrogenase; ECOG PS, Eastern Cooperative Oncology Group performance status; IPI, International Prognostic Index; PLT, platelet; LMR, lymphocyte to monocyte ratio; PLR, platelet to lymphocyte ratio; RDW ratio, red cell distribution width ratio; Hb/RDW, hemoglobin to red cell distribution width ratio; ESR, erythrocyte sedimentation rate; ALB, albumin; BMI, body max index; aaIPI, age-adjusted IPI; NCCN-IPI, National Comprehensive Cancer Network IPI P-value < 0.05.

**Table 3:** The factors associated with progression-free survival in patients with AIDS-related lymphoma (*n* = 138).

Variables	Univariate analysis		Multivariate analysis	
	HR (95% CI)	*P*	HR (95% CI)	*P*
Age > 60	1.365 (0.859–2.169)	0.188	1.554 (0.917–2.633)	0.102
Male	1.126 (0.634–1.998)	0.686		
LDH > 250	2.212 (1.222–4.005)	**0.009**	1.804 (0.776–4.193)	0.170
ECOG PS > 1	1.870 (1.216–2.875)	**0.004**	1.399 (0.771–2.536)	0.269
Ann Arbor stage III/IV	1.519 (0.989–2.332)	0.056	1.611 (0.842–3.083)	0.149
Extranodal sites > 1	1.230 (0.766–1.976)	0.391	2.232 (1.046–4.764)	**0.038**
Bulky mass ≥ 7	1.777 (1.155–2.732)	**0.009**	1.713 (1.027–2.857)	**0.039**
B symptoms (present)	1.934 (1.145–3.266)	**0.014**	1.193 (0.588–2.424)	0.625
IPI		**0.001**		
Low-intermediate	2.837 (1.558–5.164)			
High-intermediate	3.022 (1.592–5.734)			
High	3.068 (1.604–5.870)			
Prior history of HIV	1.113 (0.673–1.840)	0.676		
CD4 T cell < 300	3.022 (1.599–5.711)	**0.001**	2.328 (1.210–4.479)	**0.011**
CD4/CD8 ratio < 0.41	2.190 (1.298–3.694)	**0.003**	1.853 (1.030–3.334)	**0.040**
PLT ≥ 300	0.759 (0.487–1.183)	0.223		
LMR ≥ 3.65	0.613 (0.396–0.947)	**0.027**	0.638 (0.331–1.231)	0.180
PLR ≥ 250	1.896 (1.227–2.929)	**0.004**	1.232 (0.711–2.133)	0.457
RDW ratio ≥ 1.03	1.536 (0.990–2.383)	0.056		
Hb/RDW ≥ 8.5	0.532 (0.339–0.830)	**0.006**	0.708 (0.434–1.157)	0.168
ESR ≥ 80	1.931 (1.161–3.210)	**0.011**	1.213 (0.675–2.180)	0.520
ALB < 32	2.295 (1.484–3.547)	**<0.001**	2.225 (1.362–3.636)	**0.001**
BMI		0.186		
Overweight	1.253 (0.688–2.284)			
Emaciation	1.556 (0.808–2.996)			
aaIPI		**0.003**		
Low-intermediate	2.164 (0.830–5.638)			
High-intermediate	3.396 (1.309–8.811)			
High	4.628 (1.764–12.143)			
NCCN-IPI		**0.005**		
Low-intermediate	1.558 (0.654–3.713)			
High-intermediate	3.172 (1.329–7.571)			
High	2.822 (1.003–7.943)			

LDH, lactate dehydrogenase; ECOG PS, Eastern Cooperative Oncology Group performance status; IPI, International Prognostic Index; PLT, platelet; LMR, lymphocyte to monocyte ratio; PLR, platelet to lymphocyte ratio; RDW ratio, red cell distribution width ratio; Hb/RDW, hemoglobin to red cell distribution width ratio; ESR, erythrocyte sedimentation rate; ALB, albumin; BMI, body max index; aaIPI, age-adjusted IPI; NCCN-IPI, National Comprehensive Cancer Network IPI. P-value < 0.05.

According to the ROC curve, the optimal cutoff points of CD4 T cells, CD4/CD8 ratio, LMR, PLR, RDW ratio, Hb/RDW, ESR, and ALB were 300 cells/μl, 0.41, 3.65, 250, 1.03, 8.5, 80 mm/h, and 32 g/L, respectively. The area under the curves (AUC) for the CD4 T-cell count, CD4/CD8 ratio, LMR, PLR, RDW ratio, Hb/RDW, ESR and ALB were 0.84, 0.84, 0.61, 0.61, 0.58, 0.73, 0.63, and 0.75, respectively (**Supplementary Figure 3**).

### The CD4/CD8 Ratio as a New Inflammation Marker for Prognosis

In the present study, the novel inflammatory variables, such as CD4/CD8 ratio, LMR, PLR, and Hb/RDW, were predictive factors significantly affecting OS (all *P* < 0.01) and PFS (all *P* < 0.05) (**Tables 2**, **3**). However, only a lower CD4/CD8 ratio could predict inferior OS (HR = 2.103, 95% CI = 1.156–3.827, *P* = 0.015) and PFS (HR = 1.853, 95% CI = 1.030–3.334, *P* = 0.040) independently in the multivariate analysis, adjusted for the lymphoma- and HIV-associated parameters (**Tables 2**, **3**). Patients with a higher CD4/CD8 ratio (**Figure 2**) in the ARL cohort had significantly superior OS (*P* < 0.001) and PFS (*P* < 0.001).

**Figure 3:**
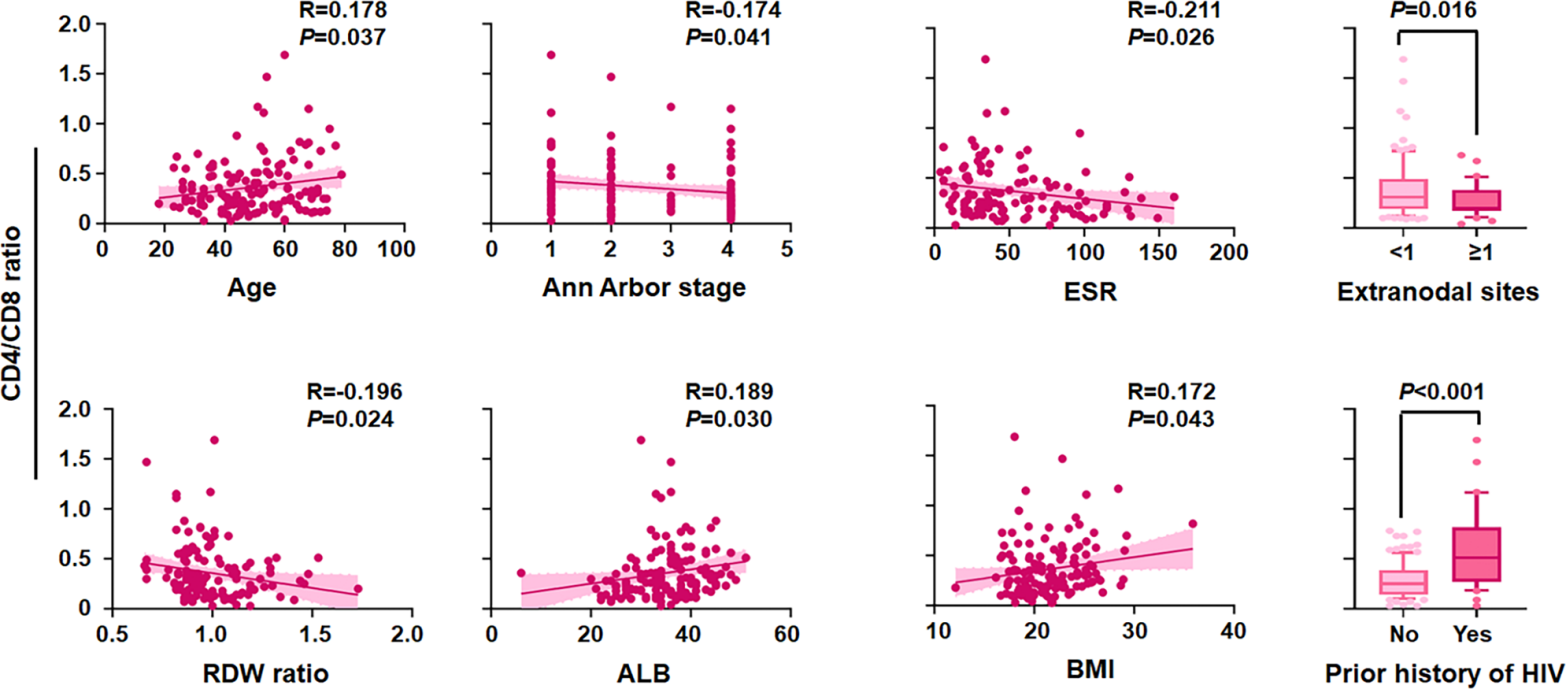
Correlation of the CD4/CD8 ratio with clinical variables. CD4/CD8 ratio level by age, Ann Arbor stage, erythrocyte sedimentation rate (ESR), extranodal sites, red cell distribution width (RDW) ratio, albumin (ALB), body max index (BMI), and prior history of HIV (*n* = 138).

Linear correlation analysis and comparative statistics demonstrated that a lower CD4/CD8 ratio was strongly correlated with adverse clinical factors (**Figure 3**), including older age (*P* = 0.037), advanced stage of Ann Arbor (*P* = 0.041), elevated ESR (*P* = 0.026), more extranodal sites (*P* = 0.016), higher RDW ratio (*P* = 0.024), hypoproteinemia (*P* = 0.030), emaciation (*P* = 0.043), and without previous history of HIV (*P* < 0.001). Furthermore, the chi-square test revealed that an inferior CD4/CD8 ratio was significantly related to older age, male sex, history of HIV, higher LDH level, poorer ECOG PS, bulky mass, presence of B symptoms, lower LMR, higher PLR, lower Hb/RDW, higher ESR, and hypoproteinemia (all *P* < 0.05) (**Table 4** and **Supplementary Table 2**).

**Table 4:** Patients’ demographics and characteristics by CD4/CD8 ratio stratification at diagnosis of AIDS-related lymphoma (*n* = 138).

Variables	CD4/CD8 ratio		
	≥0.41(*n* = 46, %)	<0.41(*n* = 92, %)	*P*
Age > 60	13 (28.26)	24 (26.87)	**0.006**
Male	31 (67.39)	81 (88.04)	**0.005**
Prior history of HIV	22 (47.83)	11 (11.96)	**0.022**
CD4 T cells < 300	19 (41.30)	83 (90.22)	0.368
LDH > 250	34 (73.91)	72 (78.26)	**0.003**
ECOG PS > 1	16 (34.78)	38 (41.30)	**0.011**
Ann Arbor stage III/IV	15 (32.61)	45 (48.91)	0.408
Extranodal sites > 1	11 (23.91)	24 (26.09)	0.413
Bulky mass (present)	18 (39.13)	43 (46.74)	**0.013**
B symptoms (present)	6 (13.04)	16 (17.39)	**0.046**
LMR < 2.33	20 (43.48)	47 (51.09)	**0.029**
PLR ≥ 250	14 (30.43)	32 (34.78)	**0.026**
RDW ratio ≥ 1.03	14 (30.43)	31 (33.70)	0.577
Hb/RDW < 8.5	25 (54.35)	49 (53.26)	**0.004**
ESR ≥ 80	7 (15.22)	22 (23.91)	**0.018**
ALB < 32	11 (23.91)	36 (39.13)	**0.036**
BMI < 18.5	11 (23.91)	18 (19.57)	0.450
IPI > 2	14 (30.43)	37 (40.22)	0.103
aaIPI > 1	20 (43.48)	51 (55.43)	**0.017**
NCCN-IPI > 3	17 (36.96)	39 (42.39)	**0.043**

LDH, lactate dehydrogenase; ECOG PS, Eastern Cooperative Oncology Group performance status; LMR, lymphocyte to monocyte ratio; PLR, platelet to lymphocyte ratio; RDW ratio, red cell distribution width ratio; Hb/RDW, hemoglobin to red cell distribution width ratio; ESR, erythrocyte sedimentation rate; ALB, albumin; BMI, body max index; IPI, International Prognostic Index; aaIPI, age-adjusted IPI; NCCN-IPI, National Comprehensive Cancer Network IPI. P-value < 0.05.

### Development of the HIV-IPI Model

**Figure 4:**
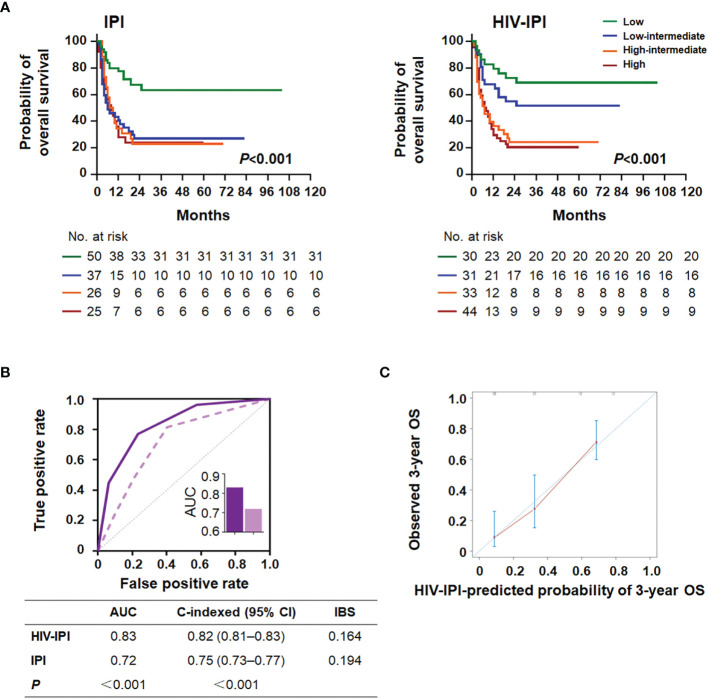
HIV-IPI risk score to predict overall survival in AIDS-related lymphoma patients. **(A)** Kaplan–Meier survival curves and log-rank *P*-values of OS according to the IPI (left) and the HIV-IPI (right) prediction models in the AIDS-related lymphoma cohort. **(B)** The receiver operating characteristic (ROC) curves, area under the curves (AUC), C-indexes, and IBS of the IPI and the HIV-IPI prognostic systems for predicting overall survival. **(C)** Calibration curve of the HIV-IPI model for predicting 3-year overall survival (*n* = 138).

The CD4/CD8 ratio and five features (age, ECOG PS, LDH, extranodal sites, and Ann Arbor stage) of the IPI score were used to design a new model, HIV-IPI, to predict the OS. The model was scored as 1 for each feature considering the hazard ratio of each category of the variables (Bento et al., 2020). The maximum score among the patients in the present study cohort was 6. After sorting the HIV-IPI model based on total score, four risk groups were classified as low-risk (score: 0–1; 21.2%), low-intermediate-risk (score: 2; 22.6%), high-intermediate risk (score: 3; 24.1%), and high-risk (score: 4–6; 32.1%) groups, which corresponded to the OS rates of 74.9% (95% CI = 59.0–90.8, *P* < 0.001), 47.2% (95% CI = 34.0–60.3, *P* = 0.003), 22.5% (95% CI = 13.0–32.0, *P* < 0.001), and 17.8% (95% CI = 11.4–24.3, *P* < 0.001), respectively. The HIV-IPI displayed better prognostic accuracy than the current IPI risk stratification model in OS estimates between the low-intermediate-risk and the high-risk groups (**Figure 4A**).

As shown in **Figure 4B**, HIV-IPI had significantly better discrimination than IPI [AUC (95% CI): HIV-IPI, 0.83 (0.77–0.89) vs. IPI, 0.72 (0.70–0.85)]. The calibration curves of the HIV-IPI model for the prediction of the 3- and 5-year OS demonstrated promising agreement between the predicted and actual results (**Figure 4C** and **Supplementary Figure 4**). The C-index for the HIV-IPI model was 0.82 (**Figure 4B**, 95% CI 0.81–0.83, *P* < 0.001), higher than that for the IPI (0.75; 95% CI 0.73–0.77, *P* < 0.001) with statistical significance (*P* < 0.001). The IBS of HIV-IPI for OS prediction demonstrated better performance than the IPI (**Figure 4B**). Collectively, these results indicated that the novel HIV-IPI outperformed the IPI and could be a useful predictive model with good discriminative ability and clinical utility for patients with AIDS in the cART era.

## Discussion

In the era of cART and immunochemotherapy, the outcome of AIDS-related lymphoma is driven by lymphoma-dependent risk factors rather than by characteristics of HIV infection in high-income countries (NOY, 2020). However, high-quality epidemiological data for ARL populations from low- and middle-income countries are scarce. Furthermore, it is uncertain whether inflammatory-based risk prediction could influence the outcome of individuals with ARL in the cART era. Thus, we conducted a multi-institutional real-world retrospective study in South China. In the current study, the CD4/CD8 ratio, an inflammatory index, proved to be an independent predictor for newly diagnosed ARL. It should be noted that we also developed HIV-IPI, a new risk stratification index, incorporating the CD4/CD8 ratio into the original IPI score, which exhibited a superior prognostic value in terms of clearly distinguishing the low-intermediate-risk group from the high-risk group. In this study, we provided new data to support the use of HIV-IPI to improve the prognostic assessment of newly diagnosed adults with ARL and to better tailor treatment objectives.

The CD4/CD8 ratio has emerged as an indicator of chronic inflammation and immunosenescence, as well as a predictor of long-term mortality in both HIV-infected and uninfected populations (Mcbride and Striker, 2017; Clifford et al., 2017; Serrano-villar et al., 2020). Firstly, the CD4/CD8 ratio is increasingly recognized as a marker of immune recovery and prognosis during HIV treatment. Early, effective, and uninterrupted cART improves the CD4/CD8 ratio (Mcbride and Striker, 2017). Previous studies collectively suggested that a lower CD4/CD8 ratio during cART is associated with a persistently higher HIV DNA (Gandhi et al., 2017), AIDS- or non-AIDS-related events and deaths (Mussini et al., 2015; Caby et al., 2021), and frailty of older HIV-infected adults (Branas et al., 2017). Second, the tumor microenvironment and the non-malignant inflammatory response play an important role at different stages of tumor development, including initiation, promotion, malignant conversion, invasion, and metastasis. The circulating CD4/CD8 ratio is an adverse prognostic factor in refractory/relapsed DLBCL–PCNSL (primary central nervous system lymphoma) (Ghesquieres et al., 2019), mantle cell lymphoma (Nygren et al., 2014), and Waldenstrom macroglobulinemia (Cao et al., 2021) in the HIV-negative population. A persistently low CD4/CD8 ratio was correlated with the risk of AIDS or non-AIDS defining cancer in PLWH efficiently treated by cART, such as Kaposi sarcoma, non-Hodgkin lymphoma, Hodgkin lymphoma, and lung cancer (Clifford et al., 2009; Sigel et al., 2017; Caby et al., 2021). However, very few studies have attempted to assess the performance of the CD4/CD8 ratio in AIDS-related lymphomas in the immunotherapy era. To our knowledge, this could be the first study to find an independently prognostic association of the CD4/CD8 ratio with mortality in populations with ARL.

The discrimination and calibration of IPI have been attenuated with time in the contemporary era. Furthermore, with the success of modern cART and viral suppression, the absolute CD4 count and HIV viral load may not accurately reflect the risks of patients because immune dysfunction persists despite normalization of the CD4 count (Mcbride and Striker, 2017). This conclusion was also confirmed in our research because the synergy of the CD4 count with IPI, ALB, ESR, and B symptoms did not have statistical significance in the development of a new prognostic index (**Supplementary Figure 6**). Therefore, the evaluation and incorporation of novel risk markers are important. Here, we integrated the CD4/CD8 ratio with the original IPI and established a new prognostic score, HIV-IPI, which was associated with significant improvements in risk reclassification and improved predictive precision in patients with ARL. Based on the proposed model, we were able to separate four distinct subpopulations precisely and a net 22.6% of ARL patients were reclassified as low-intermediate risk with a good prognosis. As more targeted therapy and immunochemotherapy options become available, decision support tools applicable to daily clinical practice are becoming increasingly important to better identify patients that may do well with conventional approaches and those who should be immediately considered for the more complex and costly new therapy (Maurer et al., 2021). The CD4/CD8 ratio is a readily available biomarker and a test routinely performed in clinical practice without expensive instruments, complex calculations, or additional costs. In the future, we expect that HIV-IPI not only will help predict survival outcomes but also will contribute to the improvement of oncological treatment.

There are limitations of the model that must be considered. First, the retrospective cohort may have a potential selection bias. Second, the external validity of the model needs to be confirmed. Third, there is likely variability in the selection of therapy and management of ARL across different centers. In countries with limited resources, adequate treatment must be provided. Fourth, given the higher proportion of low-income and middle-income patients in this real-world study, the OS for the entire series was lower than that of contemporary studies in the cART era and the rituximab era. Future evaluation and recalibration of the model in patients with ARL treated in the current era will be necessary. Nevertheless, the HIV-IPI reflects the real-life setting and is simple to apply in the clinic.

In summary, the CD4/CD8 ratio was a validated independent prognostic parameter in AIDS-related lymphoma among all the patient characteristics analyzed in our study. Although the CD4/CD8 ratio is an inexpensive, simple, and readily available clinical variable, it shows increased prognostic ability when the CD4/CD8 ratio is used in combination with the IPI in newly diagnosed ARL. We present a prognostic model, the HIV-IPI, for the risk stratification of primary ARL patients in the real-world setting for low-income and middle-income areas. The HIV-IPI is the first prognostic index to integrate the CD4/CD8 ratio into the IPI in ARL and may contribute to improving the assessment of patients with low-intermediate risk of disease progression and can inform clinical treatment decisions.

## Data Availability Statement

The raw data supporting the conclusions of this article will be made available by the authors, without undue reservation.

## Ethics Statement

This study was approved by the Institutional Ethics Committee of Nanfang Hospital (study identifier NFEC-2021-178) and was conducted in accordance with the Declaration of Helsinki. The patients/participants provided their written informed consent to participate in this study. Written informed consent was obtained from the individual(s) for the publication of any potentially identifiable images or data included in this article.

## Author Contributions

Conception and design: JP, JC, and ZX. Provision of the study materials or patients: ZX, JC, SQ, AL, and GR. Collection and assembly of data: JC, JZ, and YW. Data analysis and interpretation: JP, JC, and XL. Manuscript writing: JC. Accountable for all aspects of the work: all authors. All authors read and approved the final manuscript.

## Funding

This work was supported by the National Natural Science Foundation of China (81971949 and 82071354); the Clinical Research Program of Nanfang Hospital, Southern Medical University (2018CR026); the Natural Science Foundation of Guangdong Province (2018A030313571); and the Major Science and Technology Special Project of Nanning (20193008).

## Conflict of Interest

The authors declare that the research was conducted in the absence of any commercial or financial relationships that could be construed as a potential conflict of interest.

## Publisher’s Note

All claims expressed in this article are solely those of the authors and do not necessarily represent those of their affiliated organizations, or those of the publisher, the editors and the reviewers. Any product that may be evaluated in this article, or claim that may be made by its manufacturer, is not guaranteed or endorsed by the publisher.
