# Supplementary material for: A Novel Prognostic Score Including the CD4/CD8 for AIDS-Related Lymphoma
Source: Front Cell Infect Microbiol. 2022 Jul 6;12:919446. doi: 10.3389/fcimb.2022.919446 (PMC9299417; doi:10.3389/fcimb.2022.919446)
Supplement: Supplementary file 1 [file DataSheet_1.pdf]

Supplementary Material

Supplementary Figures

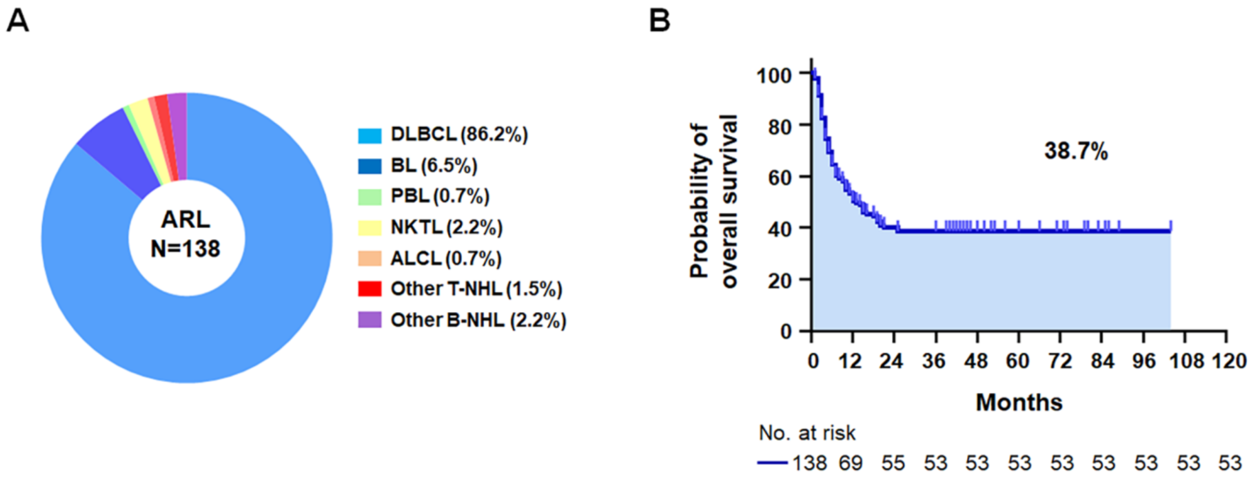

Supplementary Figure 1. Distribution of lymphoma and survival curve. (A) Distribution of AIDS-related lymphoma. (B) Overall survival in the whole cohort (n=138). DLBCL, diffuse large B-cell lymphoma; BL, Burkitt lymphoma; PBL, plasmablastic lymphoma; NKTL, natural killer/T-cell lymphoma; ALCL, anaplastic large cell lymphoma.

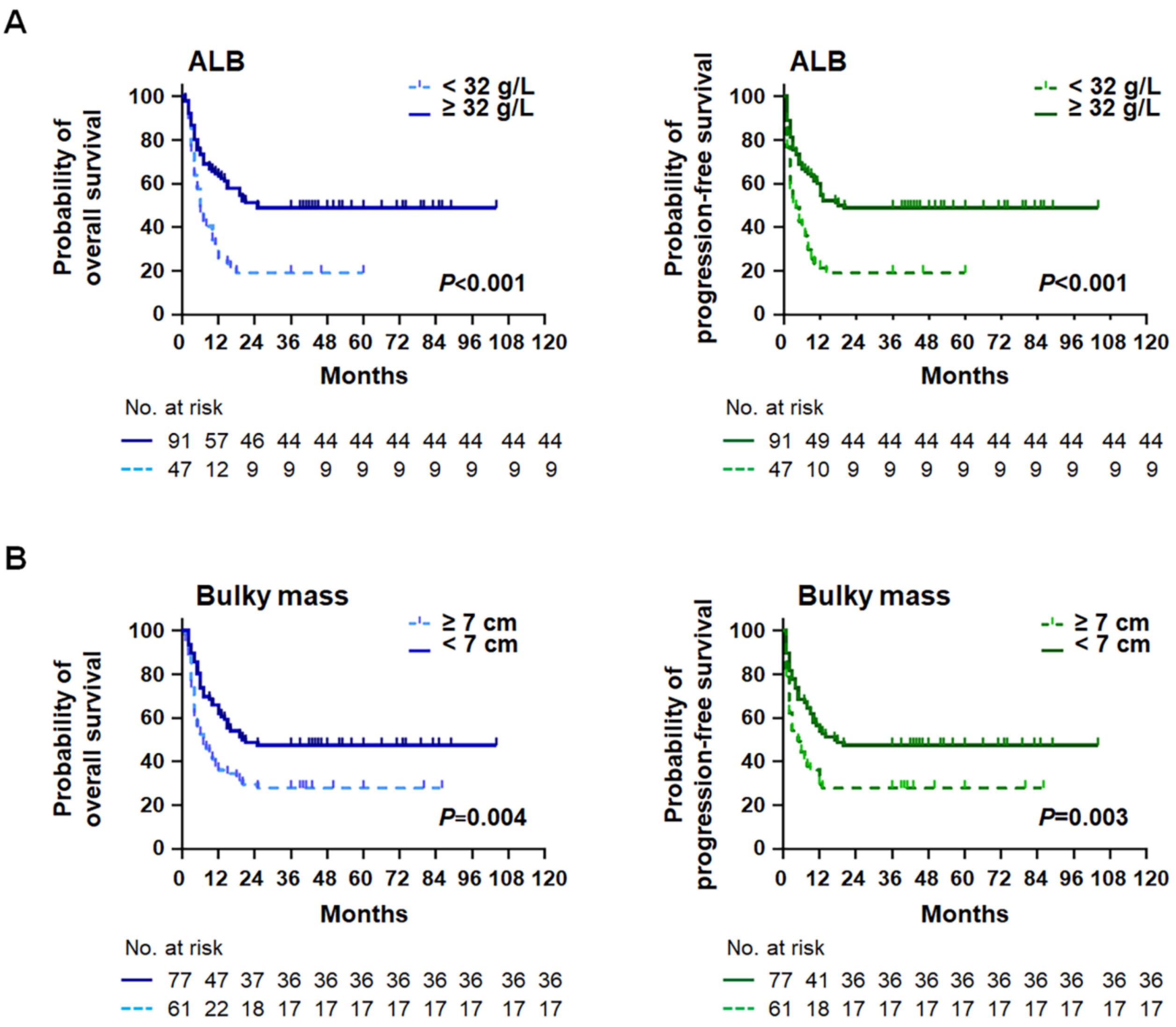

Supplementary Figure 2. Survival curves stratified by the albumin (ALB) and the bulky mass. Kaplan–Meier curves and log-rank *P* values of the ALB (A) and the bulky mass (B) stratifications for overall survival (left) and progression-free survival (right) in AIDS-related lymphoma patients (n=138).

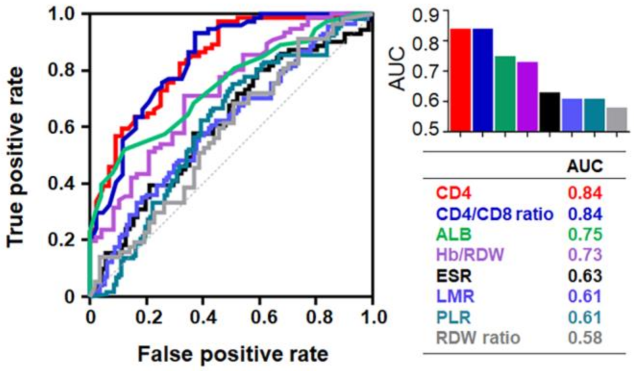

Supplementary Figure 3. The receiver operating characteristic (ROC) curves and the area under curves (AUC) of CD4+ T cell, CD4/CD8 ratio, albumin (ALB), hemoglobin to red cell distribution width ratio (Hb/RDW), erythrocyte sedimentation rate (ESR), lymphocyte to monocyte ratio (LMR), platelet to lymphocyte ratio (PLR), and RDW ratio in the AIDS-related lymphoma cohort (n=138).

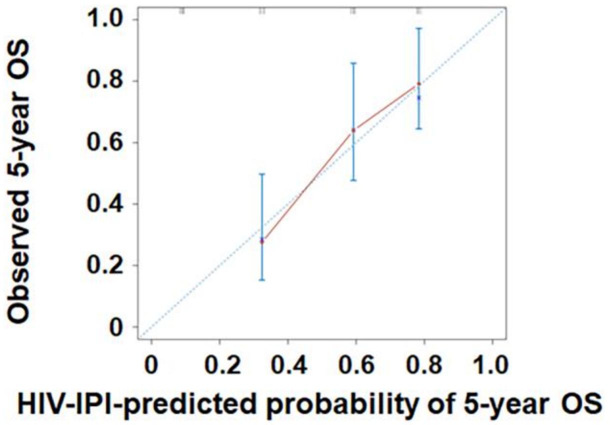

Supplementary Figure 4. Calibration curve of HIV-IPI model for predicting 5-year overall survival (n=138).

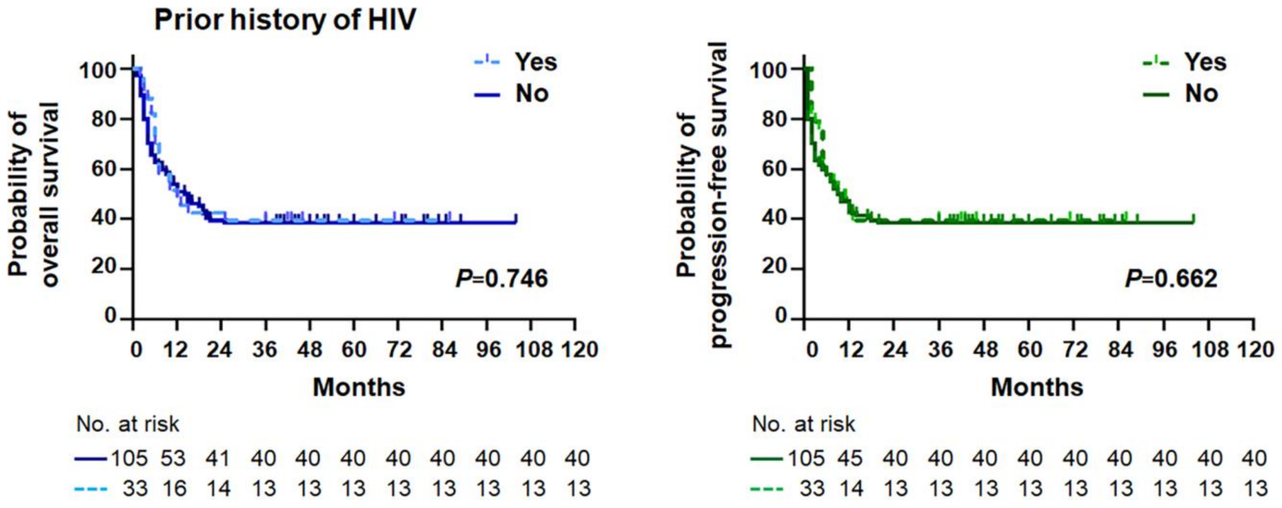

Supplementary Figure 5. Survival curves stratified by the prior history of HIV. Kaplan–Meier curve and log-rank *P* value of the prior history of HIV stratifications for overall survival (left) and progression-free survival (right) in AIDS-related lymphoma patients (n=138).

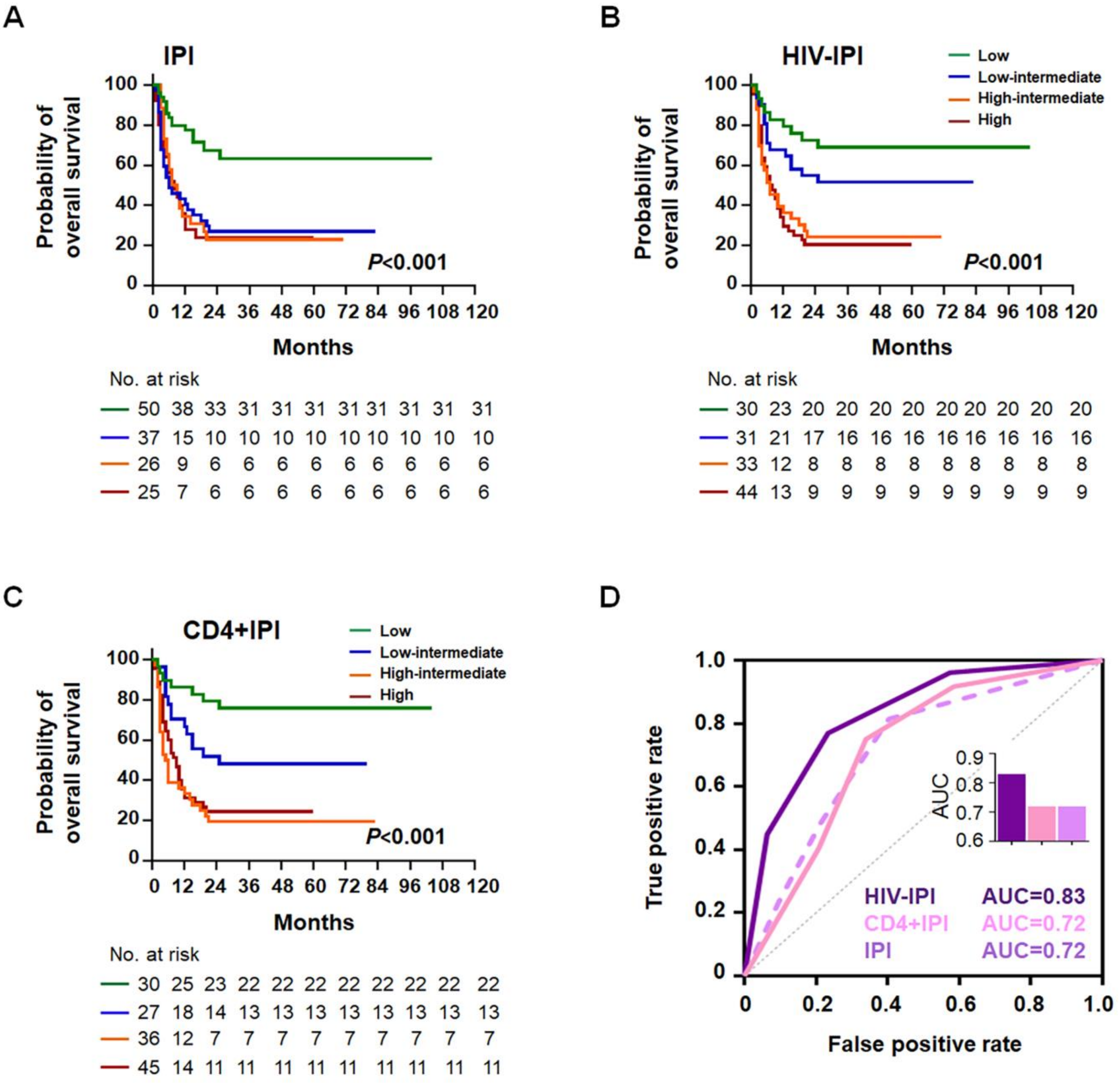

Supplementary Figure 6. HIV-IPI risk score compared with CD4+IPI risk score to predict overall survival in AIDS-related lymphoma patients. Kaplan–Meier survival curves and log-rank *P* values of OS according to the IPI (A), the HIV-IPI (B), and the CD4+IPI (C) prediction models in the AIDS-related lymphoma cohort. (D) The receiver operating characteristic (ROC) curves and the area under curves (AUC) of IPI, HIV-IPI, and CD4+IPI prognostic systems for predicting overall survival (n=138).

Supplementary Tables

Supplementary Table 1 The cell-of-origin, combination antiretroviral therapy, lymphoma treatments and outcomes (n=138).

**Subtype** (available to evaluate)	**DLBCL** (N=119)	**BL** (N=9)	**Other ARL** (N=10)	**Total** (N=138)
**Cell-of-origin phenotype**				
GCB	74 (62.2)			74 (53.6)
non-GCB	34 (28.6)			34 (24.6)
Unknown	11 (9.2)			11 (8.0)
**cART**				
TDF+3TC+EFV	71 (59.7)	4 (44.4)	8 (80.0)	83 (60.1)
AZT+3TC+EFV	6 (5.0)	2 (22.2)		8 (5.8)
AZT+3TC+NVP	1 (0.8)	1 (11.1)		2 (1.4)
TDF+3TC+LPV/r	3 (2.5)	1 (11.1)		4 (2.9)
d4T+3TC+LPV/r	2 (1.7)			2 (1.4)
3TC+LPV/r	2 (1.7)		1 (10.0)	3 (2.2)
NVP+3TC+EFV	3 (2.5)			3 (2.2)
AZT+3TC+LPV/r	1 (0.8)			1 (0.7)
d4T+3TC+EFV	2 (1.7)			2 (1.4)
FTC/TAF+DTG	1 (0.8)			1 (0.7)
TDF+3TC+DTG	3 (2.5)	1 (11.1)	1 (10.0)	5 (3.6)
TAF+3TC+DTG	1 (0.8)			1 (0.7)
E/C/F/TAF	5 (4.2)			5 (3.6)
No treatment	18 (15.1)			18 (13.0)
**Treatment received**				
CHOP	7 (5.9)		1 (10.0)	8 (5.8)
R-CHOP	2 (1.7)			2 (1.4)
DA-EPOCH	92 (77.3)	8 (88.9)	5 (50.0)	105 (76.1)
R-DA-EPOCH	5 (4.2)		1 (10.0)	6 (4.3)
GDP			2 (20.0)	2 (1.4)
Untreated	13 (10.9)	1 (11.1)	1 (10.0)	15 (10.9)
**Treatment outcomes**				
CR	29 (24.4)	1 (11.1)	5 (50.0)	35 (25.4)
PR	10 (8.4)	3 (33.3)		13 (9.4)
SD	2 (1.7)	1 (11.1)	1 (10.0)	4 (2.9)
PD				
Death	78 (65.5)	4 (44.4)	4 (40.0)	86 (62.3)

DLBCL, diffuse large B-cell lymphoma; BL, Burkitt lymphoma; ARL, AIDS-related lymphoma; GCB, germinal center B-cell-like; non-GCB, non-germinal center B-cell-like; cART, combination antiretroviral therapy; TDF, tenofovirdisoproxil; 3TC, lamividine; EFV, efavirenz; AZT, zidovudine; NVP, nevurapine; LPV/r, lopinavir/ritonavir; d4T, stavudine; DTG, dolutegravir; FTC/TAF, emtricitabine/tenofovir alafenamide; TAF, tenofovir alafenamide; E/C/F/TAF, elvitegravir/cobicistat/emtricitabine/tenofovir alafenamide; CHOP, cyclophosphamide, doxorubicin, vincristine, and prednisone; R, rituximab; DA-EPOCH, dose-adjusted etoposide, vincristine, cyclophosphamide, prednisone, and doxorubicin; GDP, gemcitabine, dexamethasone, and cisplatin; CR, complete response; PR, partial response; SD, stable disease; PD, progressive disease.

Supplementary Table 2 Patient's pathology subtypes and comorbidities by CD4/CD8 ratio stratification at diagnosis of AIDS-related lymphoma (n=138).

Variables	CD4/CD8 ratio		
	≥ 0.41 (n=46, %)	< 0.41 (n=92, %)	*P*
DLBCL	36 (78.26)	83 (90.22)	**0.004**
BL	5 (10.87)	4 (4.35)	0.524
Other ARL	5 (10.87)	5 (5.43)	0.524
Opportunistic infection	12 (27.91)	31 (72.09)	0.980
Hepatitis C	2 (25.00)	6 (75.00)	1.000
Hepatitis B	6 (40.00)	9 (60.0)	0.329
Syphilis	7 (41.18)	10 (58.82)	1.000

DLBCL, diffuse large B-cell lymphoma; BL, Burkitt lymphoma; ARL, AIDS-related lymphoma.
